# A Local and Low-Dose Chemotherapy/Autophagy-Enhancing Regimen Treatment Markedly Inhibited the Growth of Established Solid Tumors Through a Systemic Antitumor Immune Response

**DOI:** 10.3389/fonc.2021.658254

**Published:** 2021-03-30

**Authors:** Jia Yuan, Xianlin Yuan, Kunlong Wu, Junxia Gao, Liangping Li

**Affiliations:** Institute of Clinical Oncology, Research Center of Cancer Diagnosis and Therapy, and Department of Clinical Oncology, The First Affiliated Hospital of Jinan University, Guangzhou, China

**Keywords:** local chemotherapy, ICD, autophagy, neoantigens, immunotherapy

## Abstract

Chemotherapy is one of the main options for the treatment of a variety of malignant tumors. However, the severe side effects resulting from the killing of normal proliferating cells limit the application of cancer-targeting chemotherapeutic drugs. To improve the efficacy of classic systemic chemotherapy, the local delivery of high-dose chemotherapeutic drugs was developed as a method to enhance local drug concentrations and minimize systemic toxicity. Studies have demonstrated that chemotherapy is often accompanied by cancer-associated immunogenic cell death (ICD) and that autophagy is involved in the induction of ICD. To improve the efficacy of local cancer chemotherapy, we hypothesized that the local delivery of chemotherapeutic plus autophagy-enhancing agents would enhance the promotive effects of ICD on the antitumor immune response. Here, we report that a low-dose chemotherapy/autophagy enhancing regimen (CAER) not only resulted in the increased death of B16F10 and 4T1 tumor cells, but also induced higher levels of autophagy *in vitro*. Importantly, the local delivery of the CARE drugs significantly inhibited tumor growth in B16F10 and 4T1 tumor-bearing mice. Systemic antitumor T-cell immunity was observed *in vivo*, including neoantigen-specific T-cell responses. Furthermore, bioinformatic analysis of human breast cancer and melanoma tissues showed that autophagy-associated gene expression was upregulated in tumor samples. Increased autophagy and immune cell infiltration in tumor tissues were positively correlated with good prognosis of tumor patients. This work highlights a new approach to improve the effects of local chemotherapy and enhance systemic antitumor immunity.

## Introduction

After more than 100 years of development, chemotherapy has become the mainstay of treatment for a variety of cancers. Chemotherapeutic agents can inhibit cancer cell proliferation and survival through a variety of mechanisms, including by affecting the chemical structure of DNA, interrupting nucleic acid synthesis and transcription, and interfering with mitotic tubulin synthesis (1). To improve treatment efficiency, high-dose systemic chemotherapy was developed as a method to treat hematopoietic and solid tumors. However, high-dose chemotherapy also kills normal proliferating cells, thereby unavoidably leading to severe side effects, such as severe bone marrow failure and immune suppression (2, 3). Multiple infections secondary to immunosuppression resulting from routine systemic chemotherapy can reduce the prognosis and survival of cancer patients. In high-dose chemotherapy regimens, blood progenitor cell transplantation was used to reduce bone marrow- and immune-associated toxicities (4, 5). These observations highlight the need to enhance the effects of chemotherapy and reduce its side effects.

To reduce chemotherapy-related cytotoxicity, local delivery of chemotherapeutic drugs was proposed as a method to maximize local drug concentrations in the immediate tumor environment while also minimizing systemic exposure and nontarget organ toxicity (6, 7). In models of lymph node metastasis, the antitumor efficacy of local chemotherapy for regional lymph node metastasis was reported to be better than that of systemic chemotherapy, and the treatment was deemed to be safe (8). The development of interventional therapy provides a new approach for the local delivery of chemotherapeutic agents. For instance, local intra-arterial chemotherapy with a high-dose cyclophosphamide+epirubicin+5-fluorouracil (CEF) regimen for triple-negative breast cancer reduced treatment duration and increased the lesion remission rate when compared with that of the control group (routine neoadjuvant chemotherapy with CEF), while showing similar toxicity (9). Although this study highlighted the efficacy of local chemotherapy, it also revealed that chemotherapeutic agents exert a significant toxic effect, even with local application.

To reduce the side effects of regional cancer therapy, new methods must be developed that allow the synergistic eradication of cancer cells by different agents. There is increasing evidence that chemotherapy not only directly kills tumor cells, but also indirectly attacks them by activating the immune system (10). Several studies have demonstrated that chemotherapeutic agents such as anthracycline, platinum, and taxane, in addition to inducing cancer cell apoptosis, can also promote the release of tumor antigens, which further activate immune cells, and finally generate a systemic anticancer immune response (11, 12). This process is known as immunogenic cell death (ICD).

Chemotherapy-induced ICD is reported to be closely associated with autophagy (13). Autophagy is characterized by the encapsulation, processing, and degradation of various cytoplasmic components. It not only maintains cellular homeostasis in conditions of internal and external stress, but also shapes the immune response to cancer (14–16). The level of autophagy in cancer cells affects the release of several cytokines and danger signals that instruct immune effectors to induce immune responses (17–19). When autophagy is hyperactivated, tumor-derived cytoplasmic components are made available for lysosomal hydrolysis, thereby promoting antigen processing in dying tumor cells (20, 21). Additionally, highly activated autophagy can increase the secretion of adenosine triphosphate (ATP) by dying tumor cells, and ATP acts as a vital signal for the activation of dendritic cells (DCs) and cytotoxic T cells and their recruitment into the local tumor microenvironment (22, 23).

Rapamycin, one of the most commonly used autophagy-inducing agents, can promote cancer cell autophagy *via* inhibition of the mTOR pathway, thereby inhibiting tumor growth (24, 25). However, systemic rapamycin administration can also suppress the immune system by blocking mTOR on T cells, leading to reduced interleukin (IL-2) production and inhibition of T-cell proliferation, which impair antitumor immune responses (26, 27).

These observations led us to speculate that local delivery of chemotherapeutic and autophagy-enhancing drugs (chemotherapy/autophagy-enhancing regimen, CAER) might enhance the efficacy of local cancer treatment. Here, we report that a low-dose local CAER could activate autophagy and enhance autophagy-associated death *in vitro.* The local delivery of low-dose CAER drugs not only efficiently inhibit the growth of the treated malignant melanoma- and breast cancer-derived tumors, but also of the contralateral nontreated ones. Further analysis showed that the immune system was activated to target the cancer cells. This research provides a new therapeutic approach for the treatment of cancer *via* the local delivery of CAER drugs with systemic antitumor T-cell responses and reduced side effects.

## Materials and Methods

### Reagents

Rapamycin (Rap) (Sigma, USA) was dissolved in DMSO and then diluted with RPMI medium. Chemotherapeutic drugs paclitaxel (PTX) and adriamycin (ADM) were purchased from the First Affiliated Hospital of Jinan University (Guangzhou, China). PMA/Ionomycin (P/I) were purchased from Sigma, USA. The peptides for immunogenic B16F10 and 4T1 mutations were synthesized by Sangon Biotech (Shanghai, China) accordingly previous publication (**Figure S3A**). Propidium iodide (PI) was purchased from BioLegend, USA. LC3B antibody was from Cell Signaling, USA. Anti-CD3, anti-CD4, anti-CD8, and anti-FOXP3 antibodies were purchased from Abcam, Cambridge, UK. Antibodies used for flow cytometry assay were as follows: anti-CD16/32 mAb (BD Biosciences, USA), anti-CD3-PEcy5, anti-CD4-FITC, anti-CD8-FITC, anti-IFN-γ-APC, anti-TNF-α-PE, and anti-FOXP3-PE (BioLegend, USA).

### Traditional (2D) and 3D Cell Culture and *In Vitro* Cell Proliferation Assays

#### Colony Formation Assay

Cells were seeded in 12-well plates (300 cells/well) and cultured under normal *in vitro* culture conditions (2D). After five days of incubation, B16F10 and 4T1 cells were either vehicle-treated or treated with low-dose of single chemotherapy drugs (2.5 μg/mL PTX or 0.05 μg/mL ADM) for two days, or with combination of two drugs as following: the same low-dose of chemotherapeutic drugs for 12 h, followed by treatment with 0.014 μg/mL rapamycin (15 nM) for another 36 h. The medium was changed every three to four days. After two weeks, cells were stained with 0.1% crystal violet in methanol for 15 min, and the number of colonies (containing 50 or more cells) was visualized and quantified by light microscopy (CKX31, OLYMPUS, Japan).

#### Spheroid Formation and Autophagic Cell Death Staining Assay

A total of 600 B16F10 and 4T1 cells/well were seeded in ultra-low attachment 96-well plates in RPMI 1640/DMEM to establish spheroid cultures (3D). After three days, the cells were treated with vehicle or chemotherapeutic drugs (5 μg/mL PTX or 0.1 μg/mL ADM) for 6–8 h followed by treatment with 0.023 μg/mL rapamycin (25 nM) for another 16–24 h. Finally, the diameter of each spheroid was measured after one week. The spheroids were stained with PI to determine the level of autophagic cell death.

### Autophagy Assays

#### Monodansylcadaverine (MDC) Staining for Autophagy

The entire dynamic autophagic process (autophagic flux) can be measured using the autofluorescent dye MDC, which specifically marks autophagic vacuoles. In brief, 2000 cells of B16F10 or 4T1 were seeded in a 96-well plate in RPMI 1640/DMEM culture medium and incubated for two days at 37°C. Then, the cells were treated with vehicle or a low dose of chemotherapeutic drugs (5 μg/mL PTX or 0.1 μg/mL ADM) for 6–8 h, followed by treatment with rapamycin (25 nM) for another 16–24 h. The cells were then stained with MDC (Solarbio, USA) and observed using fluorescence microscopy.

#### LC3 Immunofluorescence and WB Assay

To measure the level of autophagy, cells were treated as described in section 2.3.1. The cells were then fixed in cold absolute methanol and blocked with 1% BSA in PBST buffer (PBS with 0.1% Tween 20) for 1 h and incubated with the primary antibody against LC3B overnight at 4°C. The cells were subsequently incubated with a fluorochrome-conjugated secondary antibody diluted in blocking buffer for 1 h at room temperature in the dark. Finally, the stained samples were mounted in Prolong Diamond Antifade with DAPI (Invitrogen, USA). Fluorescence images were acquired and processed using ImageJ software.

Quantified tumor cell lysates protein prepared from B16F10 or 4T1 cells were loaded onto SDS-PAGE and transferred to a PVDF membranes. The primary antibodies used were LC3 (1:1000) and GAPDH (1:1000). Primary antibodies were incubated overnight at 4°C followed by washing and the application of secondary HRP-conjugated antibody. The immunoreactive bands were visualized with a chemiluminescent substrate.

#### Autophagosome Ultrastructure Assay

The autophagosomes of B16F10 and 4T1 cells were directly identified by transmission electron microscopy. Cells were fixed and embedded. Thin sections (90 nm) were examined at 80 kV with a JEOL 1200EX transmission electron microscope. Autophagosomes were defined as double-membrane vacuoles (0.1–1.0 μm).

### Mouse Models and *In Vivo* Local Treatment

Mice were purchased from Guangdong Animal Center (Guangzhou, China) and were kept in specific pathogen-free conditions. The animal experiment was conducted at Jinan University (Guangzhou, China), complying with the national guidelines for the care and use of laboratory animal.

The melanoma model was established with six–eight-week-old female C57BL/6 mice. 3×10^4^ B16F10 cells were implanted subcutaneously on both sides of abdomen (left implanted tumor used for local treatment injection, right one for observation of tumor growth). Five days after inoculation, mice were randomly distributed into various groups. Local treatment schedule: PTX (10μg, 10μg and 15μg) were intratumorally injected into tumors at left side on day 5, 9 and 11; rapamycin (2.5μg, 2.5μg and 5μg) were injected intratumorally at left side on day 7, 9 and 11. The last two injections were carried out as the following scheme: PTX (10μg and 15μg) and rapamycin (2.5μg and 5μg) were separately injected into tumors in 6-8h interval on day 9 and 11.

The mouse breast carcinoma model was established with six–eight-week-old female BALB/c mice. BALB/c mice were implanted subcutaneously with 3×10^5^ 4T1 cells on both sides of abdomen. Seven days after inoculation, BALB/c mice were randomly distributed into various groups. Local treatment schedule: ADM (0.4μg, 0.4μg and 0.8μg) were injected intratumorally into tumor of left side on day 7, 11 and 13. Rapamycin (2.5μg, 2.5μg and 5μg) were injected intratumorally on day 9, 11 and 13. The last two injections were as following: ADM (0.4μg and 0.8μg) and rapamycin (2.5μg and 5μg) were separately injected into tumors in 6–8h interval on day 11 and 13.

C57BL/6 or BALB/c mice were killed on day 21 or day 30 after tumor cells inoculation. Tumor volume was measured with a caliper and calculated using the formula (A×B^2^)/2 (A as the largest and B the smallest diameter of the tumor).

### *In Vitro* Generation of Mature Bone Marrow-Derived Dendritic Cells (BMDCs) and Antigen Stimulation of Lymphocytes

BMDCs were prepared from tibias and femurs of six–eight-week-old healthy female C57BL/6 and BALB/c mice. Bone marrow cells were passed through a 40 μm cell strainer (BD Falcon). After centrifugation at 200 × *g* for 3 min, red blood cells were lysed with 2 mL of ACK lysis buffer (Biolegend, USA) for 10 min. The remaining cells were then plated in six-well plates at a density of 1×10^6^ cells/mL in RPMI 1640 medium supplemented with 10% FBS, 20 ng/mL mouse granulocyte macrophage colony-stimulating factor (GM-CSF) (Peprotech, USA), 10 ng/mL mouse IL-4 (Peprotech), and 50 μM β-mercaptoethanol (Sigma, USA). Half of the medium was replaced with an equal volume of fresh medium containing the cytokines every three days. Seven days after the initial plating, non- and semi-adherent cells were harvested and regarded as mature BMDCs, and were loaded with mitomycin C treated tumor cells (1:5) or long peptides (20 μg/mL) for two days.

Spleens were harvested under sterile conditions from C57BL/6 and BALB/c mice with local treatment and T cells were isolated as responder cells using a 40 μm cell strainer. Mixing splenocytes with the antigen loaded BMDCs (10:1). After 72–96 h of incubation, cell clusters were observed and imaged under light microscope (CKX31, OLYMPUS, Japan).

### Immunological Assays

#### Immunohistochemistry (IHC) Assay

Tumors were fixed with 4% paraformaldehyde. Tumor tissue slides were stained with antibodies against CD3, CD4, CD8 and FOXP3. IHC images were acquired using light microscope. CD4, CD8 and FOXP3 stained areas were quantified within manually pre-defined tumor regions *via* computerized image analysis Imagine J software.

#### IFN-γ ELISpot Assay

5×10^5^ splenocytes were added into anti-IFN-γ coated multiscreen 96-well plates. BMDCs loaded with tumor cells (1:5) or 20 μg/mL peptides were added as reactants. After 12–16h of incubation at 37°C, cytokine secretion was detected with an anti-IFN-γ antibody (ELISpot) Kit (DAKEWE, Shenzhen). IFN-γ spots were scanned and quantified using a ELISPOT analyzer and ImmunoSpot Professional Software (CTL-ImmunoSpot^®^ S6 FluoroSpot, USA). P/I (500 ng/mL PMA and 1 μg/mL ionomycin) were used for positive control.

#### Flow Cytometric Assay

Single cell samples were pre-incubated with anti-CD16/32 mAb for 15 minutes at 4°C, and then stained for 30 minutes at 4°C with various combinations of fluorochrome-conjugated antibodies: anti-CD3-PEcy5, anti-CD4-FITC and anti-CD8-FITC. For intracellular cytokine staining, mouse immune cells were restimulated with antigen-loaded DC in complete RPMI 1640 media and 50 IU/mL recombinant IL-2 (Peprotech, USA) at 37°C. After an hour, 1 × GolgiStop and 1 × GolgiPlug (BD Biosciences, USA) were incubated for another 4–8 hours at 37°C. Then, cells were permeabilized using a Fixation and Permeabilization Kit (BD, USA) and stained with antibodies: anti-IFN-γ-APC, anti-TNF-α-PE and anti-FOXP3-PE. Stained cells were acquired using a flow cytometer (FASC Canto II, BD, USA) and BD FACSDiva software. The data were further analyzed with FlowJo software (version 10.4).

### Data Mining From Public Databases

The online website Gene Expression Profiling Interactive Analysis (GEPIA; available at http://gepia.cancer-pku.cn/index.html) was used to investigate the differential expression of autophagy-related 5 (ATG5) in breast invasive carcinoma (BRCA) and skin cutaneous melanoma (SKCM) tissues and explore the prognostic value of the differential expression of ATG5 in BRCA and SKCM patients. The Tumor IMmune Estimation Resource (TIMER) database—a comprehensive resource for the automatic analysis and visualization of the association between immune infiltration levels and a series of variables (https://cistrome.shinyapps.io/timer/)—was used to explore the correlation between ATG5 expression and the abundance of six types of immune cells (CD4^+^ T cells, CD8^+^ T cells, B cells, neutrophils, DCs, and macrophages) in BRCA and SKCM.

### Statistical Analysis

All values were expressed as means ± SD. Statistically significant differences among individual treatments and the corresponding control groups were determined by the Student’s *t*-test or analysis of variance (ANOVA). The Kaplan–Meier method was used to assess animal survival times and the log-rank test was used to test differences between groups of mice. Experiments were independently repeated at least three times. All analyses were carried out using GraphPad Prism 5. A *P*-value <0.05 was considered to be statistically significant.

## Results

### Rapamycin Enhanced the Inhibitory Effects of Chemotherapeutic Drugs on B16F10/4T1 Cancer Cell Proliferation and Cell Death in Both 2D and 3D Cultures

**Figure 1:**
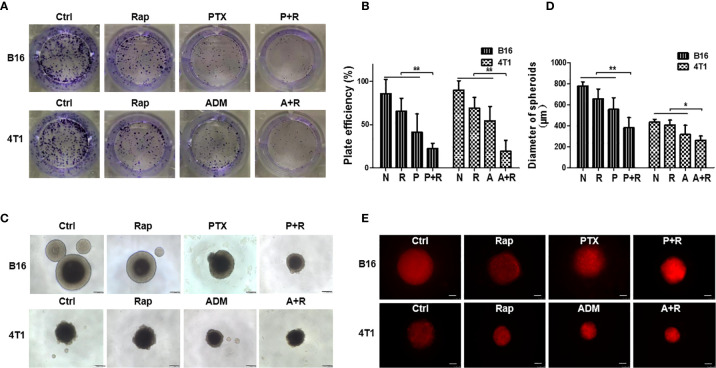
Rapamycin enhanced the inhibitory effects of chemotherapy on B16F10/4T1 cancer cell proliferation and induction of cell death. **(A)** Colony-formation assay for the proliferation of B16F10 and 4T1 cells under different drug treatments. **(B)** Graph of the statistical analysis of the plating efficiency. **(C)** Spheroid formation was used to evaluate tumor cell proliferation in 3D cultures (scale bars: 200 μm). **(D)** Graph of the statistical analysis of spheroid diameter. **(E)** Autophagy-associated death of cancer cell spheroids was measured by propidium iodide (PI) staining and imaged using red fluorescence (scale bars: 250 μm). B16: B16F10. Ctrl: negative control group. Rap: rapamycin. P+R, PTX+Rap; A+R, ADM+Rap. Bars and error bars represent means ± SD, respectively, of three independent experiments. *P < 0.05, **P < 0.01 by Student’s *t*-test.

To explore the antitumor effects of the classic chemotherapeutic plus autophagy-enhancing agents *in vitro*, we utilized two classic cancer cell lines, namely, B16F10, a malignant melanoma cell line, and 4T1, a triple-negative breast cancer cell line. The IC50 values of PTX for B16F10 cells, of ADM for 4T1 cells, and of rapamycin for both cell lines were determined by MTT assays (**Figures S1A, B**). Low dose chemotherapy (2.5–5.0 μg/mL PTX or 0.05–0.10 μg/mL ADM) was then used to investigate the synergistic effects of the chemotherapeutic drugs with rapamycin. First, a contact-dependent plate clone-formation assay was performed to assess the proliferative ability of the cancer cells. We measured cancer cell proliferation *in vitro* after treatment with the single drugs or with a CAER drugs (**Figures 1A, B**) and found that, at a low concentration (15 nM), rapamycin alone only slightly inhibited the proliferation of the two types of cancer cells (approximately 25% inhibition). A low dosage of each chemotherapeutic agent (5 μg/mL PTX or 0.1 μg/mL ADM) led to an approximately 50% inhibition of cancer cell proliferation. As expected, a combination of low-dose chemotherapy plus rapamycin (PTX+rapamycin for B16F10 cells and ADM+rapamycin for 4T1 cells) elicited a synergistic inhibitory effect on cancer growth (approximately 80%), indicating that a low-dose CAER could inhibit the proliferative ability of B16F10 and 4T1 cells.

To mimic *in vivo* conditions, we treated cancer cell spheroids generated under 3D-culture conditions with the double concentrations of the drugs (10 μg/mL PTX or 0.2 μg/mL ADM). Cancer cells treated with the CAER formed smaller spheroids than cells in the negative control group or those treated with each drug alone (**Figures 1C, D**). Additionally, the PI staining results indicated that cell death was higher in the B16F10- and 4T1-derived spheroids treated with PTX+rapamycin or ADM+rapamycin, respectively than the negative control group or those treated with each drug alone (**Figure 1E**). Together, these results demonstrated that the CAER synergistically inhibited B16F10/4T1 cell proliferation and enhanced cell death *in vitro*.

### CAER Treatment Increased the Activation of Autophagy

**Figure 2:**
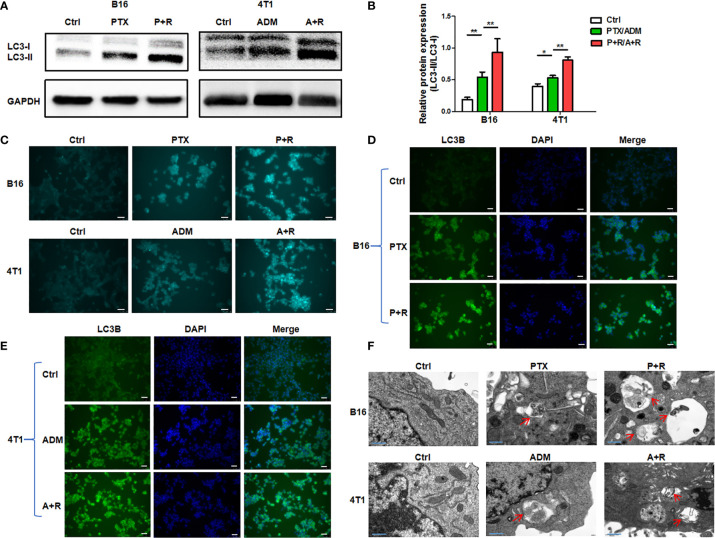
The induction of hyperautophagy by low-dose CAER treatment. **(A)** The autophagy marker LC3-II/LC3-I was detected in B16F10 (left panel) and 4T1 (right panel) cells using WB. Cells were incubated with a low concentration of paclitaxel (PTX; 5 μg/mL) or adriamycin (ADM; 0.1 μg/mL) for 6–8 h and then with rapamycin (25 nM) for 16–24h **(B)** Graph of the statistical analysis of WB. **(C)** The autophagic flux in B16F10 and 4T1 cells was detected by monodansylcadaverine (MDC) assay. (scale bars: 25 μm). **(D, E)** The autophagy marker LC3B was detected in B16F10 cells using immunofluorescence. The treatment was the same as in **Figure 1A** (scale bars: 25 μm). **(F)** Transmission electron micrographs of autophagosomes/autolysosomes induced by the chemotherapeutic agents and rapamycin in B16F10 and 4T1 cells (scale bars: 0.4 μm). *P < 0.05, **P < 0.01 by Student’s t-test.

To clarify whether treatment using the CAER could hyperactivate autophagy in cancer cells, we treated B16F10 and 4T1 cells with 5 μg/mL PTX or 0.1 μg/mL ADM, respectively, for 6–8 h, and then with rapamycin (25 nM) for another 16–24 h. Firstly, WB showed that the relative protein expression of LC3-II/LC3-I was significantly increased after CAER treatment. The ratio of LC3-II to LC3-I is proportional to the level of autophagy (**Figures 2A, B**). Secondly, we measured the autophagic flux in the cancer cells by MDC assay. Autophagic flux staining was nearly undetectable in the control cells, and was also weak in cells treated with the individual drugs. In comparison, cells treated with the CAER drugs showed substantially stronger autophagic flux staining (**Figure 2C**). Thirdly, we measured the expression level of the autophagy-specific marker LC3B by immunofluorescence staining, and found that LC3B staining was strongest in cells treated with the CAER, followed by those treated with PTX or ADM alone, and then by the control, normal medium-treated cells (**Figures 2D, E**). Finally, observation by electron microscopy showed that CAER-treated B16F10 and 4T1 cells contained more autophagosomes than those treated with either PTX or ADM alone or that seen in control cells (**Figure 2F**). Taken together, these findings showed that the CAER could induce the hyperactivation of autophagy in B16F10 and 4T1 cells.

### Local Treatment With the CAER Strongly Inhibited the Growth of B16F10 Cell-/4T1 Cell-Derived Tumors *In Vivo*

**Figure 3:**
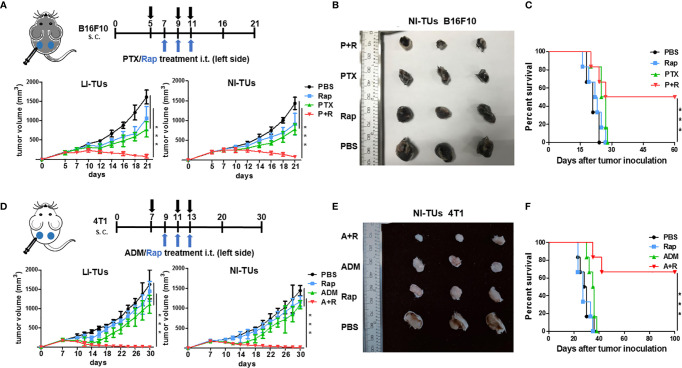
The inhibition of *in vivo* tumor growth using a local CAER treatment. **(A)** The treatment scheme (upper panel) and *in vivo* growth curve (lower panel) of B16F10 cell-derived tumors. **(B)** Representative images of B16F10 cell-derived noninjected tumors on day 16. **(C)** The survival curves for C57BL/6 mice bearing B16F10 cell-derived tumors. **(D)** The treatment scheme (upper panel) and *in vivo* growth curve (lower panel) of 4T1 cell-derived tumors. **(E)** Representative images of 4T1 cell-derived noninjected tumors on day 20. **(F)** The survival curves for BALB/c mice bearing 4T1 cell-derived tumors. LI-TUs, locally injected tumors; NI-TUs, noninjected tumors; *n* = 5–6 mice/group. ***P < 0.001 by Student’s *t*-test.

To test whether the low-dose CAER could inhibit tumor growth *in vivo*, we established tumor models in C57BL/6 or BALB/c mice by injecting B16F10 or 4T1 cells into both sides of the abdomen of the animals and then delivered the drugs locally into one tumor at left side (**Figures 3A, D**). Analysis of the growth curve of the locally injected tumors (LI-TUs) indicated that local monotherapy with PTX for B16F10 cell-derived tumors (**Figure 3A**) or ADM for 4T1-derived tumors (**Figure 3D**) exerted a significantly greater antitumor than rapamycin treatment alone. However, treatment using the CAER conferred greater antitumor activity than PTX, ADM, or rapamycin treatment alone.

**Figure 4:**
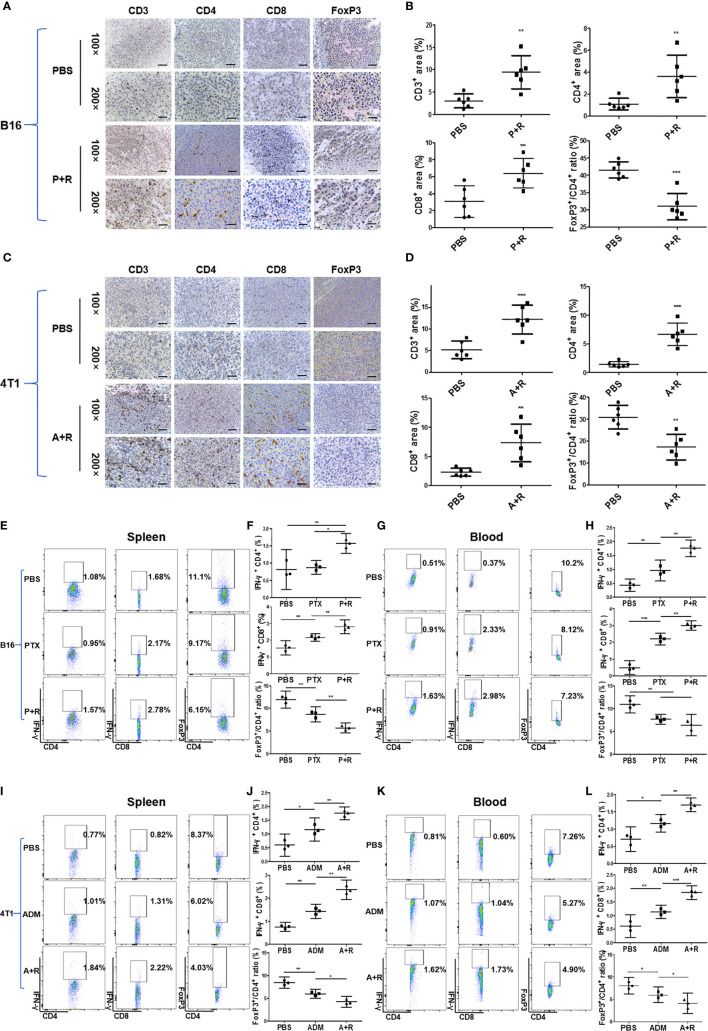
Immunological analysis of the local and systemic effects after local CAER treatment. **(A, C)** Immunological analysis of treated tumors in B16F10 and 4T1 mouse models. Representative images showing immunohistochemical (IHC) staining of CD3, CD4, CD8, and FOXP3 in tumor samples on day 16 (B16F10 cells) or day 20 (4T1 cells). Phosphate-buffered saline (PBS) was used as control (scale bars: 50 µm). **(B, D)** Graph of the statistical analysis of the IHC staining results in the B16F10 mouse model and the 4T1 mouse model. **(E, G)** Flow cytometric analysis of the spleen and peripheral blood in B16F10 tumor models. Flow cytometric analysis of the percentages of IFN-γ-producing CD4^+^ T cells, CD8^+^ T cells, and FoxP3^+^ T cells in the spleen and peripheral blood of C57BL/6 tumor model mice under the different treatment groups (PBS, PTX, and P+R groups) on day 16. **(F, H)** Graph of the statistical analysis of the flow cytometry results for B16F10 tumor models. **(I, K)** Flow cytometric analysis of the spleen and peripheral blood in 4T1 tumor models. Spleen and peripheral blood were analyzed by flow cytometry to determine the percentages of IFN-γ-producing CD4^+^ T cells, CD8^+^ T cells, and FoxP3^+^ T cells in BALB/c tumor model mice of the different treatment groups (PBS, ADM, and A+R groups) on day 20. **(J, L)** Graph of the statistical analysis of the flow cytometry results for 4T1 tumor models. *P < 0.05, **P < 0.01, ***P < 0.001 by Student’s *t*-test.

Surprisingly, we observed that the growth of the contralateral, noninjected tumors (NI-TUs) was also inhibited in mice of the groups treated with the CAER [PTX+rapamycin for B16F10 cell-derived tumors (**Figures 3A, B**) and ADM+rapamycin for 4T1 cell-derived tumors (**Figures 3D, E**)]. Survival curve analysis indicated that the CAER also improved the survival of mice in both the B16F10 (**Figure 3C**) and 4T1 (**Figure 3F**) tumor model groups. These data indicated that the local application of the CAER to LI-TUs can activate a systemic antitumor immune response and inhibit the growth of distantly contralateral NI-TUs.

### Systemic Anti-Tumor Immune Response Induced by Local CAER Treatment

To investigate the immunological changes after local CAER treatment, we analyzed the local (tumor) and systemic (spleen and blood) immune responses in the mouse tumor models using immunohistochemical (IHC) and flow cytometric assays. IHC staining showed that infiltration by CD3^+^, CD4^+^, and CD8^+^ T cells was significantly greater in B16F10-derived tumor tissues administered the CAER than control group or single drug group (**Figure 4A**). T cell infiltration increased two- to three- fold: [PTX+rapamycin *vs*. control: approximately 10% *vs*. 3% of CD3^+^, 4% *vs*. 1% of CD4^+^, and 6% *vs*. 3% of CD8^+^ T cells (**Figure 4B**)]. FoxP3^+^ T cells (Tregs) significantly reduced: 41% *vs*. 32% of CD4^+^ T cells from analysis of **Figure 4A**. Similar results were obtained for the 4T1-derived tumor models (**Figures 4C, D**).

To analyze the systemic immune response after local treatments, we measured the levels of IFN-γ secreted by T cells in the spleen and peripheral blood of B16F10 tumor model mice through flow cytometry. Spleen data showed that CD4^+^ T cells and CD8^+^ T cells from the CAER treatment groups (PTX+rapamycin) secreted greater amounts of IFN-γ than those in the PBS or PTX monotherapy groups (**Figure 4E**). CD8^+^ T cells (1.10% increasing) responded more obvious than CD4^+^ T cells (0.49% increasing). Meanwhile, Tregs decreased 4.95% in the CAER group of the spleen (**Figure 4F**). In blood analysis for B16F10, CD8^+^ T cells (2.61% increasing) were triggered more obviously than CD4^+^ T cells (1.12% increasing), but Tregs decreased 2.97% in the CAER therapy group (**Figures 4G, H**). Similar results could be found in 4T1 tumor models (**Figures 4I–L**): the CAER (A+R) treatment promoted the activation of more T cells or inhibition of Tregs in tumor, spleen and blood of 4T1 tumor-bearing BALB/c mice.

### Local CAER Treatment Induced a Tumor Antigen-Specific T-Cell Response *In Vivo*

**Figure 5:**
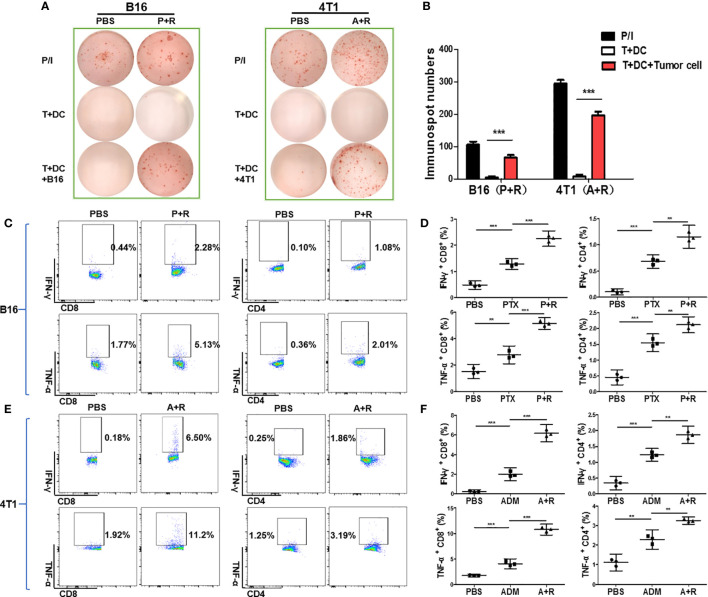
Local CAER treatment increased the number of tumor-reactive T cells *in vivo*. **(A)** ELISpot analysis for immune responses to T cells under the different treatments (PBS, PTX/ADM, and P+R/A+R). The immune responses of splenocytes isolated from B16F10 tumor-bearing C57BL/6 mice or 4T1 tumor-bearing BABL/c mice of the different treatment groups were tested by ELISpot for the recognition of B16F10 and 4T1 tumor cells. **(B)** Graph of the statistical analysis of ImmunoSpot numbers. **(C, E)** Flow cytometric analysis of CD4/CD8 surface and intracellular cytokine (IFN-γ and TNF-α) staining to detect the activation of splenic T cells incubated with B16F10 or 4T1 tumor cells. **(D, F)** Graph of the statistical analysis of the flow cytometry results in the B16F10 and 4T1 models. P/I, PMA+ionomycin (positive control). **P < 0.01, ***P < 0.001 by Student’s *t*-test.

Our results demonstrated that local CAER treatment could trigger systemic antitumor immunity in two cancer models in mice with different genetic backgrounds. To further elucidate the mechanisms underlying these immunological changes, we performed *ex vivo* stimulation to determine whether CAER treatment could activate tumor-reactive T cells in tumors derived from B16F10 or 4T1 cells. Splenocytes from B16F10-bearing C57BL/6 mice or 4T1-bearing BALB/c mice treated with the local CAER were stimulated with mature DCs loaded with intact B16F10 or 4T1 tumor cells treated with mitomycin C. ELISpot assay data showed that splenocytes from mice treated using the local CAER exhibited a stronger response to tumor cell-derived antigens than those isolated from mice in the control group (**Figures 5A, B**).

To analyze the T-cell responses in the different groups in more detail, we used flow cytometry to analyze intracellular cytokine levels (IFN-γ and TNF-α staining). The data showed that the secretion of both cytokines was significantly upregulated in the PTX+rapamycin and ADM+rapamycin treatment groups when compared with controls. The incubation of tumor cells with splenic T cells derived from B16F10-bearing mice treated with the local CAER led to the activation of a greater number of tumor antigen-specific CD4^+^ T cells and CD8^+^ T cells than that with T cells in the other treatment groups (**Figures 5C, D**). CD8^+^ T cell responded in two-fold more than CD4+ T cell (IFN-γ secreted CD8^+^ T cells increase to 1.84%, while CD4^+^ T cells to 0.98%; the secretion of TNF-α was increased to 3.36% of CD8^+^ T cells, while 1.65% of CD4^+^ T cells). Similarly, both of CD4^+^ T cells and CD8^+^ T cells were activated in the CAER treatment group of 4T1 tumor-bearing BALB/c mice and CD8+ T cells responded dominantly (IFN-γ secreted CD8^+^ T cells increase to 6.32%, while CD4^+^ T cells to 1.61%; the secretion of TNF-α was increased to 9.28% in CD8^+^ T cells, while to 1.94% in CD4^+^ T cells) (**Figures 5E, F**). These results showed that, compared with the other treatments, local CAER administration activated a greater number of tumor-reactive T cells *in vivo*.

### Local CAER Treatment Induced Neoantigen-Specific T-Cell Responses

**Figure 6:**
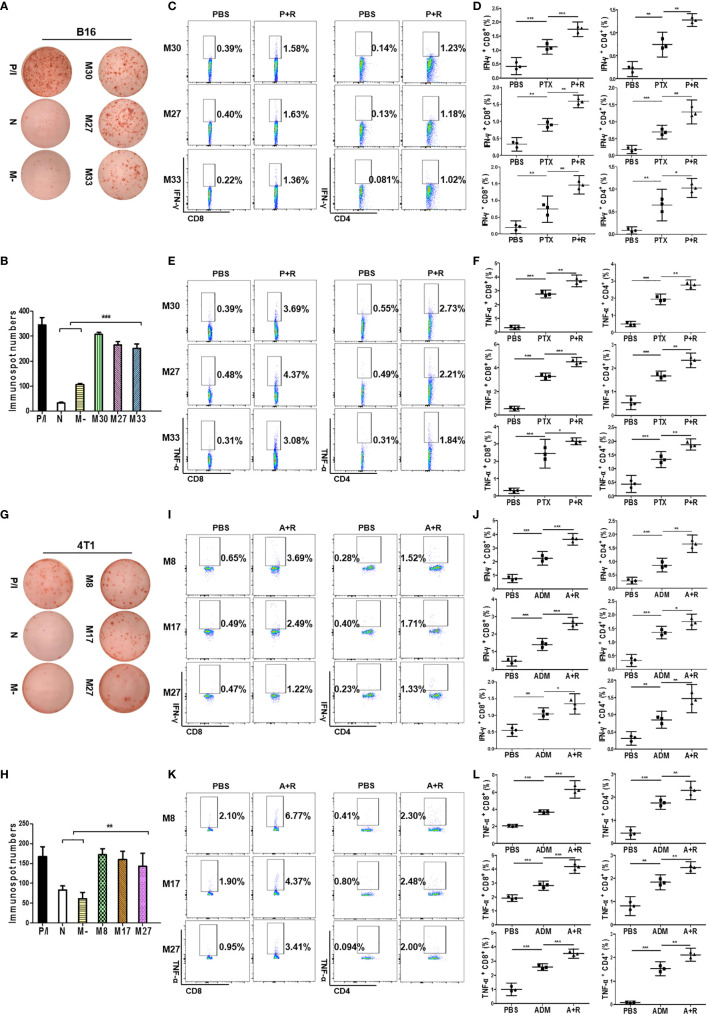
Local CAER Treatment induce neoantigen-specific T-cell responses. **(A)** IFN-γ ELISpot assay for T cell immune responses targeting neoantigens. Splenocytes from B16F10 cell-derived, tumor-bearing C57BL/6 mice under the different treatments (PBS, PTX, and P+R) were stimulated by BMDCs plus neoantigen peptides (M30, M27 and M33) and detected by IFN-γ ELISpot assay. **(B)** Graph of the statistical analysis of ImmunoSpot numbers in the B16F10 model. **(C, E)** Flow cytometric analysis of intracellular cytokine (IFN-γ and TNF-α) secretion levels during neoantigen-specific T cell immune responses in the B16F10 model. **(D, F)** Graph of the statistical analysis of the flow cytometry results in the B16F10 model. **(G)** IFN-γ ELISpot assay for T cell immune responses targeting neoantigens. Splenocytes from 4T1 cell-derived, tumor-bearing BABL/c mice under the different treatments (PBS, ADM, and A+R) were stimulated by BMDCs plus neoantigen peptides (M8, M17, M27) and detected by IFN-γ ELISpot assay. **(H)** Graph of the statistical analysis of ImmunoSpot numbers in the 4T1 model. **(I, K)** Flow cytometric analysis of intracellular cytokine (IFN-γ and TNF-α) secretion levels during neoantigen-specific T cell immune responses in the 4T1 model. **(J, L)** Graph of the statistical analysis of the flow cytometry results in the 4T1 model. N, no peptide; M-, negative peptide. *P < 0.05, **P < 0.01, ***P < 0.001 by Student’s *t*-test.

Because tumor-specific neoantigens are key targets for antitumor immune responses, we next sought to verify whether the CAER could activate neoantigen-specific T cells. We previously synthesized and prescreened mutant neoantigen peptides from B16F10 and 4T1 cells (28) (**Figure S3A**). Three mutant peptides (B16-M27, B16-M30, and B16-M33) derived from B16F10 cells, and three (4T1-M8, 4T1-M17, and 4T1-M27) from 4T1 cells, were used for the subsequent experiments. Splenocytes from C57BL/6 mice bearing B16F10 cell-derived tumors and administered the CAER regimen were incubated with peptide-loaded (B16-M27, B16-M30, or B16-M33) DCs to evaluate the neoantigen-reactive T cell immune response. ELISpot assays showed that all the peptide-loaded DCs stimulated T cells to produce IFN-γ (**Figures 6A, B**). To determine the composition of the neoantigen-reactive T-cell population, we next analyzed intracellular cytokine staining by flow cytometry. The analysis demonstrated that, when incubated with neoantigen peptides, splenic T cells isolated from C57BL/6 mice bearing B16F10 cell-derived tumors and treated with CAER generated more activated CD4^+^ and CD8^+^ T cells than those isolated from mice in the other treatment groups (**Figures 6C–F**). B16-M30 treatment induced increases in IFN-γ/TNF-α levels of 1.19%/3.30% in CD8^+^ T cells and 1.09%/2.18% in CD4^+^ T cells; for B16-M27, the increases were 1.23%/3.89% and 1.05%/1.72%, respectively; and for B16-M33, the respective increases were 1.14%/2.77% and 0.939%/1.53%.

Similar results were obtained for 4T1 cell-derived tumor-bearing BALB/c mice, further indicating that treatment with a local CAER could enhance neoantigen-specific T cell responses (4T1-M8 treatment induced increases in IFN-γ/TNF-α levels of 3.04%/4.67% in CD8^+^ T cells and 1.24%/1.89% in CD4^+^ T cells; for 4T1-M17, the increases were 2.00%/2.47% and 1.31%/1.68%, respectively; and for 4T1-M27, the respective increases were 0.75%/2.46% and 1.10%/1.91%) (**Figures 6G–L**). These results indicated that CD8^+^ T cells elicited the strongest responses to the three neoantigens from each cell line.

### Bioinformatic Analysis of the Expression of the Autophagy Biomarker ATG5 and the Relationship Between ATG5 and Immune Cell Infiltration Levels in BRCA and SKCM

**Figure 7:**
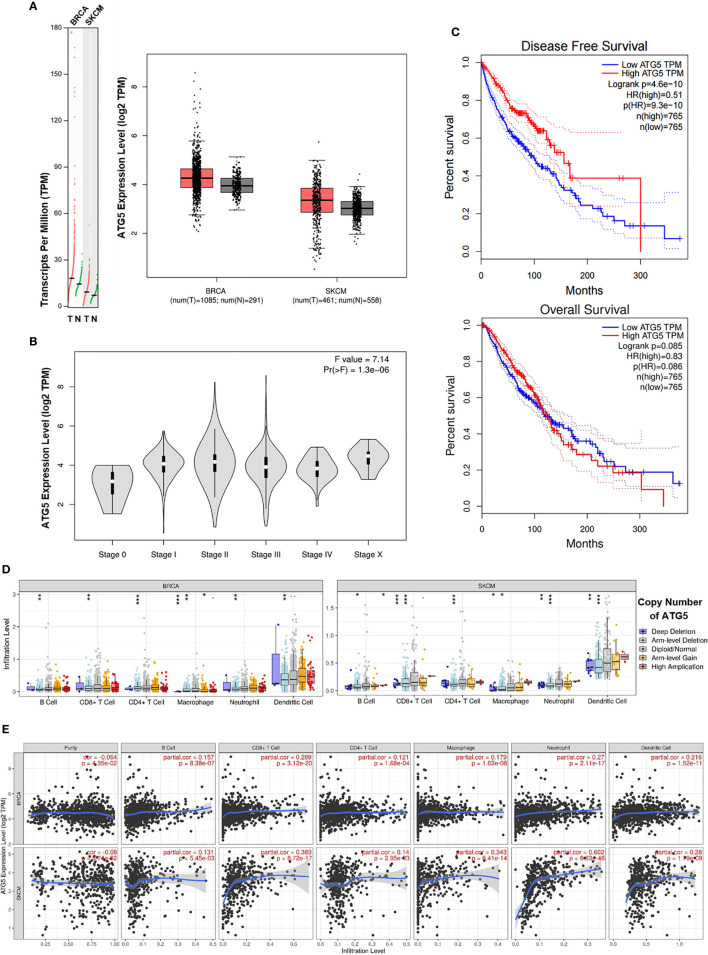
Bioinformatic analysis of the expression of the autophagy biomarker ATG5 and the relationship between ATG5 and immune infiltration levels in BRCA and SKCM. **(A)** The ATG5 mRNA (left panel) and protein expression (right panel) levels were higher in BRCA and SKCM tissue than in normal tissue. **(B)** ATG5 expression levels in different clinical stages in BRCA and SKCM patients. **(C)** Kaplan–Meier survival curves of disease-free survival (upper panel) and overall survival (lower panel) for high and low ATG5 expression groups in BRCA and SKCM patients. **(D)** A comparison of immune infiltration levels among BRCA (left panel) and SKCM (right panel) tumors with different ATG5 gene copy number status. **(E)** The correlation between ATG5 expression and the abundance of six types of immune cells (B cells, CD8^+^ T cells, CD4^+^ T cells, macrophages, neutrophils, and dendritic cells) in BRCA (upper panel) and SKCM tissues (lower panel). BRCA, breast invasive carcinoma; SKCM, skin cutaneous melanoma. *P < 0.05, **P < 0.01, ***P < 0.001.

To assess the status of autophagy in human breast carcinoma (BRCA) and skin cutaneous melanoma (SKCM), we first analyzed the levels of autophagy in these human cancer types based on data obtained from the GEPIA website. ATG5, a key autophagy-related gene, is reported to be positively correlated with the level of autophagy (29–31). We found that the ATG5 expression level was markedly higher in BRCA and SKCM tissues than in matched normal tissues (**Figure 7A**). Furthermore, the expression level of ATG5 was increased with increasing clinical staging in BRCA and SKCM patients (**Figure 7B**). Next, we generated Kaplan–Meier plots to investigate the prognostic value of the level of autophagy in patients with BRCA and SKCM, with the results showing that high ATG5 expression levels were correlated with better disease-free survival (DFS) (**Figure 7C** upper panel) and forepart of overall survival (OS) (**Figure 7C** lower panel). These data indicated that early-stage BRCA and SKCM might respond better to low-dose CAER treatment. In addition, higher concentrations of CAER drugs might be required to treat late-stage BRCA and SKCM.

Subsequently, we employed the TIMER database to determine whether any correlation existed between ATG5 expression and immune cell infiltration. TIMER-based analysis indicated that infiltration by CD4^+^/CD8^+^ T cells and DCs was significantly reduced with arm-level deletion of ATG5 in BRCA and SKCM tissues (**Figure 7D**). We next focused on the correlations between ATG5 expression and the infiltration of six immune cell types in BRCA and SKCM using the TIMER database. As shown in **Figure 7E**, ATG5 expression was markedly correlated with B cell, CD8^+^ T cell (maximum correlation), CD4^+^ T cell, macrophage, neutrophil, and DC infiltration. Meanwhile, the correlations between ATG5 expression and the abundance of the six types of immune cells in different types of BRCA and SKCM were also studied (**Figures S4A, B**). Finally, due to the promise associated with immune checkpoint inhibitor therapy for cancer treatment, we further determined the association between ATG5 and PD-1 and CTLA-4 expression (**Figure S4C**), and found that there was a negative correlation between ATG5 and PD-1 (*r* = −0.069, *P* < 0.05) as well as between ATG5 and CTLA-4 (*r* = −0.04, *P* = 0.11) in BRCA and SKCM tumors. Combined, these data indicated that the level of autophagy was correlated with the prognosis of BRCA and SKCM patients and immune cell infiltration in BRCA and SKCM tumors.

## Discussion

Cancer chemotherapy-associated immune suppression remains a key drawback for cancer treatment. The suppression of immunity is associated with the dosage and route of chemotherapeutic drug administration. High-dose systemic chemotherapy always destroys bone marrow stem cells and immune cells and results in severe immune suppression (2). To reduce chemotherapy-related cytotoxicity, the local delivery of chemotherapeutic drugs was proposed and tested in clinical trials. The results of these trials revealed that this approach could reduce systemic toxicity and prolong the survival of cancer patients (7, 32). However, local high-dose chemotherapy still destroys a large number of tumor-reactive T cells that infiltrate the regional tumor tissue and normal tissue cells nearby, which further promotes the progression of the disease (3).

To avoid these potentially harmful effects, we designed a local cancer CAER treatment that combined low-dose chemotherapy with low-dose autophagy therapy. *In vitro*, we demonstrated that low-dose autophagy-inducing rapamycin (25 nM) could greatly enhance cancer-inhibiting effects of low-dose chemotherapeutic drug administration. Additionally, this regimen also resulted in higher rates of cell death, as determined by *in vitro* PI-staining assays.

We successfully applied this strategy in animal models, which led to the complete eradication of well-established tumors derived from both B16F10 and 4T1 cells. Tumor growth was significantly inhibited in mice from the two models treated with low-dose CAER, while mouse survival was significantly improved. Additionally, we observed a large number of infiltrated CD3^+^ T cells in LI-TUs and NI-TUs. Importantly, local CAER administration also led to the eradication of distant NI-TUs. These observations indicate that local drug injection has a therapeutic effect in remote regions, and even the whole body, through systemic antitumor immune responses. We further analyzed the tumor, spleen, and peripheral blood and found increased numbers of CD3^+^ T (both CD4^+^ and CD8^+^ T cells) in the combination-treatment groups, whereas the numbers of CD4^+^ FoxP3^+^ T cells were decreased. These results suggested that treatment using local administration of a combination of CAER can induce a strong systemic antitumor immune response.

Tumor antigens, especially neoantigens, are key targets for anticancer immunotherapy (33–35). The local administration of CAER agents to tumor cells can lead not only to increased tumor cell death but also to the release of a larger amount of tumor antigens, including neoantigens, for antigen presentation (36–38). With our local therapy regimen, the tumors were quickly eradicated, concomitant with the infiltration of a large number of T cells. To determine whether neoantigen-specific T cells were activated by this local treatment, we examined the neoantigen-specific immune response based on previous neoepitope-related studies in these two tumor models by next-generation sequencing (28). We found that a local CAER could activate neoantigen-specific CD4^+^ and CD8^+^ T cells, as evidenced by flow cytometric analysis of intracellular IFN-γ and TNF-α staining. These experiments demonstrated that our strategy could lead to the activation of T cells that target these neoantigens, thereby triggering systemic immune responses.

Our animal model data demonstrated that the hyperactivation of autophagy in cancer cells was associated with systemic antitumor immune responses. To assess the relationship between autophagy and immunity in human cancers, we measured the expression levels of autophagy-related genes in BRCA and SKCM through bioinformatics tools. The results indicated that the level of autophagy was higher in early-stage BRCA and SKCM tissue, while Kaplan–Meier plots demonstrated that higher levels of autophagy were correlated with more favorable DFS and better forepart of OS among BRCA and SKCM patients. When exploring the possible reasons behind the clinical prognosis, we further discovered that immune cell infiltration, especially that by CD8^+^ T cells, was markedly correlated with the level of autophagy in BRCA and SKCM tumors. This suggest that high levels of autophagy in tumor samples, together with T-cell infiltration, can serve as a predictor of better tumor prognosis and has positive clinical significance.

The applicability of this research is mainly reflected in the following two aspects. First, although neoantigen-based vaccines have shown promising efficacy, the application of personalized neoantigen-based vaccines remains limited owing to multiple factors, including an unavoidable original sample error, tumor heterogeneity, predictive accuracy, and high cost (39). Treatment using our CAER could induce ICD and lead to the release of a higher amount of endogenous neoantigens, thereby enhancing tumor immunogenicity and triggering systemic immune responses without the need for sequencing to identify mutant neoantigen sequences. These advantages render our method more convenient than some of those previously reported (40, 41). Furthermore, combining our regimen with other immunomodulators is expected to further improve its efficacy. Indeed, robust antitumor responses were achieved with local chemotherapy combined with a CTLA-4 blockade in murine models of melanoma and prostate cancer (42). These observations suggest that treatment using a local CAER combined with immune checkpoint inhibitors or immunoadjuvants may represent a promising option for cancer immunotherapy in the future (43–46). In this sense, our study broadens the range of neoantigen-related treatments and may provide a new option for the treatment of patients with advanced malignant tumors.

In summary, the key finding of this study was that cancer treatment using local administration of CAER generated an effective local and systemic antitumor response in mice. Our work revealed that hyperactivated autophagy can boost chemotherapy-induced antitumor immunity, especially the activation of neoantigen-specific T cells. Accordingly, local CAER therapy not only reduced systemic toxicity, but also modified the tumor microenvironment, thereby inducing anticancer immunity.

## Data Availability Statement

The original contributions presented in the study are included in the article/**Supplementary Material**. Further inquiries can be directed to the corresponding author.

## Ethics Statement

The animal study was reviewed and approved by Jinan University Experimental Animal Center.

## Author Contributions

JY and XY performed the experiments and wrote the paper. KW and JG performed data processing and statistical analysis. LL designed, proposed, and supervised this project. All authors contributed to the article and approved the submitted version.

## Funding

This project was supported by National Key Research & Development Projects (2016YFC1303404 and 2016YFA0101103) of the Ministry of Science and Technology, and Guangdong Science and Technology Project (2014A020211006) from the Guangdong Science and Technology Innovation Commission, of the People’s Republic of China.

## Conflict of Interest

The authors declare that the research was conducted in the absence of any commercial or financial relationships that could be construed as a potential conflict of interest.

## References

[1] DeVita VT Chu E. A History of Cancer Chemotherapy. Cancer Res (2008) 68:8643–53. 10.1158/0008-5472.CAN-07-6611 18974103

[2] van der Wall E Beijnen JH Rodenhuis S. High-dose chemotherapy regimens for solid tumors. Cancer Treat Rev (1995) 21:105–32. 10.1016/0305-7372(95)90023-3 7758003

[3] Hunter WL Burt HM Machan L. Local delivery of chemotherapy: a supplement to existing cancer treatments. Adv Drug Deliv Rev (1997) 26:199–207. 10.1016/S0169-409X(97)00035-5 10837543

[4] Sukru O Ugur S Nuri K Fikret A Taner D. An overview of high dose chemotherapy with autologous stem cell rescue for germ cell tumors in current practice. J B U ON (2017) 22:306–11. 28534349

[5] Jeremy W Le Deley M-C Uta D Le Teuff G Bernadette B Nathalie G. High-Dose Chemotherapy and Blood Autologous Stem-Cell Rescue Compared with Standard Chemotherapy in Localized High-Risk Ewing Sarcoma: Results of Euro-E.W.I.N.G.99 and Ewing-2008. J Clin Oncol (2018) 36:JCO2018782516. 10.1200/JCO.2018.78.2516 PMC6209090 30188789

[6] Krukiewicz K Zak JK. Biomaterial-based regional chemotherapy: Local anticancer drug delivery to enhance chemotherapy and minimize its side-effects. Mat Sci Eng C (2016) 62:927–42. 10.1016/j.msec.2016.01.063 26952500

[7] Falke LL van Vuuren Stefan H Filis K Farshad R Nguyen TQ Veldhuis JG. Local therapeutic efficacy with reduced systemic side effects by rapamycin-loaded subcapsular microspheres. Biomaterials (2015) 42:151–60. 10.1016/j.biomaterials.2014.11.042 25542803

[8] Heegon K Hyeonjeong H Moonkyoung J Junhee H Ho PJ. Management of lymph node metastasis via local chemotherapy can prevent distant metastasis and improve survival in mice. J Controlled Release (2021) 329:847–57. 10.1016/j.jconrel.2020.10.016 33065097

[9] He J Wang X Guan H Chen W Wang M Wu H. Clinical efficacy of local targeted chemotherapy for triple-negative breast cancer. Radiol Oncol (2011) 45:123–8. 10.2478/v10019-011-0014-7 PMC3423732 22933945

[10] Nowak AK Lake RA Marzo AL Scott B Heath WR Collins EJ. Induction of tumor cell apoptosis in vivo increases tumor antigen cross-presentation, cross-priming rather than cross-tolerizing host tumor-specific CD8 T cells. J Immunol (Baltimore Md.: 1950) (2003) 170:4905–13. 10.4049/jimmunol.170.10.4905 12734333

[11] Yi-Jun W Rochelle F Jian Y Lin Z. Immunogenic effects of chemotherapy-induced tumor cell death. Genes Dis (2018) 5:194–203. 10.1016/j.gendis.2018.05.003 30320184 PMC6176216

[12] Niels H Sara M Matthias K Inka Z Axel B Anna S. Localization and density of immune cells in the invasive margin of human colorectal cancer liver metastases are prognostic for response to chemotherapy. Cancer Res (2011) 71:5670–7. 10.1158/0008-5472.CAN-11-0268 21846824

[13] Martins I Michaud M Sukkurwala AQ Adjemian S Ma Y Shen S. Premortem autophagy determines the immunogenicity of chemotherapy-induced cancer cell death. Autophagy (2012) 8:413–15. 10.4161/auto.19009 22361584

[14] Zhenyu Z Elsa S Michael K. Autophagy, Inflammation, and Immunity: A Troika Governing Cancer and Its Treatment. Cell (2016) 166:288–98. 10.1016/j.cell.2016.05.051 PMC4947210 27419869

[15] Ma Y Galluzzi L Zitvogel L Kroemer G. Autophagy and Cellular Immune Responses. Immunity (2013) 39:211–27. 10.1016/j.immuni.2013.07.017 23973220

[16] Kuballa P Nolte WM Castoreno AB Xavier RJ. Autophagy and the immune system. Annu Rev Immunol (2012) 30:611–46. 10.1146/annurev-immunol-020711-074948 22449030

[17] Michaud M Martins I Sukkurwala AQ Adjemian S Ma Y Pellegatti P. Autophagy-Dependent Anticancer Immune Responses Induced by Chemotherapeutic Agents in Mice. Science (2011) 334:1573–7. 10.1126/science.1208347 22174255

[18] Michaud M Xie X Pedro JMB Zitvogel L White E Kroemer G. An autophagy-dependent anticancer immune response determines the efficacy of melanoma chemotherapy. OncoImmunology (2014) 3:e944047. 10.4161/21624011.2014.944047 25610726 PMC4292732

[19] Hernandez C Huebener P Schwabe RF. Damage-associated molecular patterns in cancer: a double-edged sword. Oncogene (2016) 35:5931–41. 10.1038/onc.2016.104 PMC5119456 27086930

[20] Federico P Manuel BPJ Lorenzo G Guido K. Autophagy in natural and therapy-driven anticancer immunosurveillance. Autophagy (2017) 13:2163–70. 10.1080/15548627.2017.1310356 PMC5788556 28598229

[21] van de Ven R Hilton TL Hu HM Dubay CJ Haley D Paustian C. Autophagosome-based strategy to monitor apparent tumor-specific CD8 T cells in patients with prostate cancer. OncoImmunology (2018) 7:e1466766. 10.1080/2162402X.2018.1466766 30524883 PMC6279418

[22] Shaun M Dudek-Peric AM Garg AD Heleen R Seyma D Van Eygen S. An autophagy-driven pathway of ATP secretion supports the aggressive phenotype of BRAF^V600E^ inhibitor-resistant metastatic melanoma cells. Autophagy (2017) 13:1512–27. 10.1080/15548627.2017.1332550 PMC5612289 28722539

[23] Fader CM Aguilera MO Colombo MI. ATP is released from autophagic vesicles to the extracellular space in a VAMP7-dependent manner. Autophagy (2012) 8:1741–56. 10.4161/auto.21858 PMC3541285 22951367

[24] Srivastava RK Utley A Shrikant PA. Rapamycin. OncoImmunology (2014) 1:1189–90. 10.4161/onci.20663 PMC3494641 23170275

[25] Jung CH Ro S Cao J Otto NM Kim D. mTOR regulation of autophagy. FEBS Lett (2010) 584:1287–95. 10.1016/j.febslet.2010.01.017 PMC2846630 20083114

[26] Chaoul N Fayolle C Desrues B Oberkampf M Tang A Ladant D. Rapamycin Impairs Antitumor CD8+ T-cell Responses and Vaccine-Induced Tumor Eradication. Cancer Res (2015) 75:3279–91. 10.1158/0008-5472.CAN-15-0454 26122844

[27] Li Q Rao R Vazzana J Goedegebuure P Odunsi K Gillanders W. Regulating Mammalian Target of Rapamycin To Tune Vaccination-Induced CD8. J Immunol (2012) 188:3080–7. 10.4049/jimmunol.1103365 PMC3311730 22379028

[28] Sebastian K Mathias V van de Roemer N Mustafa D Martin L Jan D. Mutant MHC class II epitopes drive therapeutic immune responses to cancer. Nature (2015) 520:692–6. 10.1038/nature14426 PMC4838069 25901682

[29] Cassidy LD Young AR Pérez-Mancera PA Nimmervoll B Jaulim A Chen HC. A novel Atg5 -shRNA mouse model enables temporal control of Autophagy in vivo. Autophagy (2018) 14:1256–66. 10.1080/15548627.2018.1458172 PMC6103714 29999454

[30] Arbogast F Arnold J Hammann P Kuhn L Chicher J Murera D. ATG5 is required for B cell polarization and presentation of particulate antigens. Autophagy (2019) 15:280–94. 10.1080/15548627.2018.1516327 PMC6333460 30196744

[31] Pyo J Yoo S Ahn H Nah J Hong S Kam T. Overexpression of Atg5 in mice activates autophagy and extends lifespan. Nat Commun (2013) 4:604–12. 10.1038/ncomms3300 PMC3753544 23939249

[32] Bohatyrewicz A Karaczun M Kotrych D Ziętek P Kołodziej Ł Jurewicz A. Solitary breast cancer metastasis to pelvic bone treated with a unique method of surgery combined with local doxorubicin administration. Contemp Oncol (2017) 21:306–10. 10.5114/wo.2017.72402 PMC5798423 29416438

[33] Kishton RJ Lynn RC Restifo NP. Strength in Numbers: Identifying Neoantigen Targets for Cancer Immunotherapy. Cell (2020) 183:591–3. 10.1016/j.cell.2020.10.011 33125888

[34] Jiang T Shi T Zhang H Hu J Song Y Wei J. Tumor neoantigens: from basic research to clinical applications. J Hematol Oncol (2019) 12:263–70. 10.1186/s13045-019-0787-5 PMC6731555 31492199

[35] Sarah C. Neoantigen vaccine proven safe and immunogenic. Nat Rev Drug Discovery (2020) 19:838. 10.1038/d41573-020-00194-x 33139898

[36] Chen H Yang G Xiao J Zheng L You L Zhang T. Neoantigen-based immunotherapy in pancreatic ductal adenocarcinoma (PDAC). Cancer Lett (2020) 490:12–9. 10.1016/j.canlet.2020.06.011 32590021

[37] Jiao HX Lei MX Li Y Quan WY Yong P Wei WX. Progress in Neoantigen Targeted Cancer Immunotherapies. Front Cell Dev Biol (2020) 8:728. 10.3389/fcell.2020.00728 32850843 PMC7406675

[38] Xuhui W Man L Kebai R Chunyu X Jianping L Qianwen Y. On-Demand Autophagy Cascade Amplification Nanoparticles Precisely Enhanced Oxaliplatin-Induced Cancer Immunotherapy. Adv Mat (Deerfield Beach Fla.) (2020) 32:e2002160. 10.1002/adma.202002160 32596861

[39] Lancaster EM David Jablons Kratz JR. Applications of Next-Generation Sequencing in Neoantigen Prediction and Cancer Vaccine Development. Genet Test Mol Biomark (2020) 24:59–66. 10.1089/gtmb.2018.0211 30907630

[40] Li Q Ding Z. The Ways of Isolating Neoantigen-Specific T Cells. Front Oncol (2020) 10:1347. 10.3389/fonc.2020.01347 32850430 PMC7431921

[41] Sachie H Emiko N Hiroko B Hiroko M Wataru M Takako N. Neoantigen prediction in human breast cancer using RNA sequencing data. Cancer Sci (2020) 112:465–75. 10.1111/CAS.14720 PMC7780012 33155341

[42] Ariyan CE Brady MS Siegelbaum RH Hu J Bello DM Rand J. Robust Antitumor Responses Result from Local Chemotherapy and CTLA-4 Blockade. Cancer Immunol Res (2018) 6:189–200. 10.1158/2326-6066.CIR-17-0356 29339377 PMC6857638

[43] ChiPing H ChunChie W ChihRong S. Combination of novel intravesical xenogeneic urothelial cell immunotherapy and chemotherapy enhances anti-tumor efficacy in preclinical murine bladder tumor models. Cancer Immunol Immunother (2020) 6:1–15. 10.1007/s00262-020-02775-6 PMC8053151 33156394

[44] Davern M Lysaght J. Cooperation between chemotherapy and immunotherapy in gastroesophageal cancers. Cancer Lett (2020) 495:89–99. 10.1016/J.CANLET.2020.09.014 32950619

[45] Yadong W Xiaoying Y Xu T Ziqi J Zhongxing B Lei C. Neoadjuvant immunotherapy plus chemotherapy achieved pathologic complete response in stage IIIB lung adenocarcinoma harbored EGFR G779F: a case report. Ann Palliat Med (2020) 9:4339–45. 10.21037/apm-20-1692 33183013

[46] Wu D Fan Y Yan H Li D Zhao Z Chen X. Oxidation-sensitive polymeric nanocarrier-mediated cascade PDT chemotherapy for synergistic cancer therapy and potentiated checkpoint blockade immunotherapy. Chem Eng J (2021) 404:126481–. 10.1016/J.CEJ.2020.126481

