# Supplementary material for: A Local and Low-Dose Chemotherapy/Autophagy-Enhancing Regimen Treatment Markedly Inhibited the Growth of Established Solid Tumors Through a Systemic Antitumor Immune Response
Source: Front Oncol. 2021 Mar 30;11:658254. doi: 10.3389/fonc.2021.658254 (PMC8042230; doi:10.3389/fonc.2021.658254)
Supplement: Supplementary file 1 [file DataSheet_1.docx]

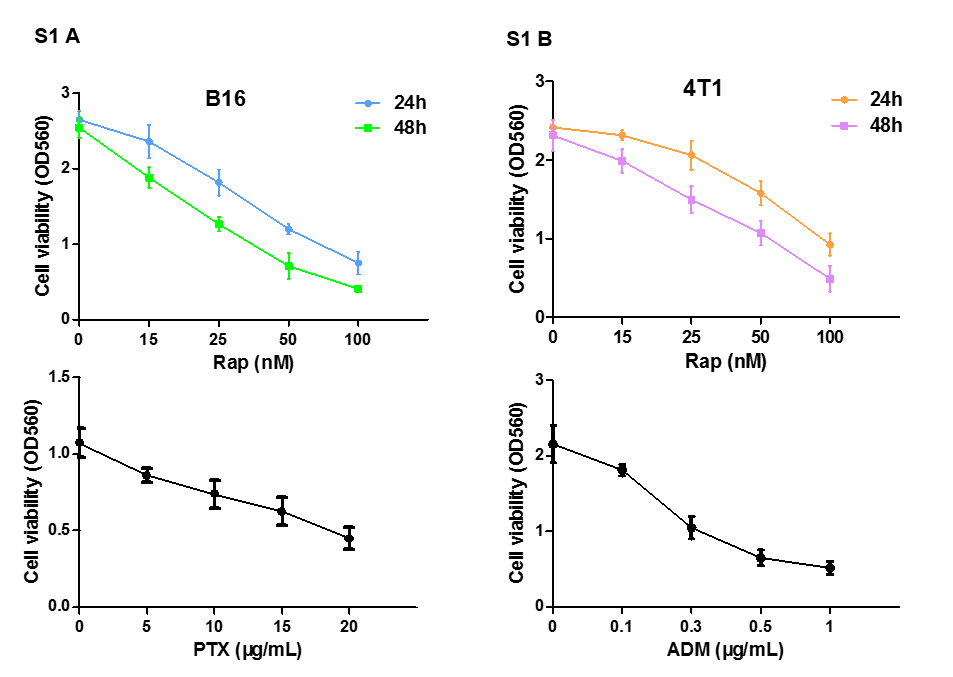


**FIGURE S1 |** Drug sensitivity of rapamycin and chemotherapeutic drugs for B16F10 and 4T1 cells *in vitro.* **(A)** IC50 of rapamycin and PTX for B16F10 cells were measured by MTT. **(B)** IC50 of rapamycin and ADM for 4T1 cells were measured by MTT.


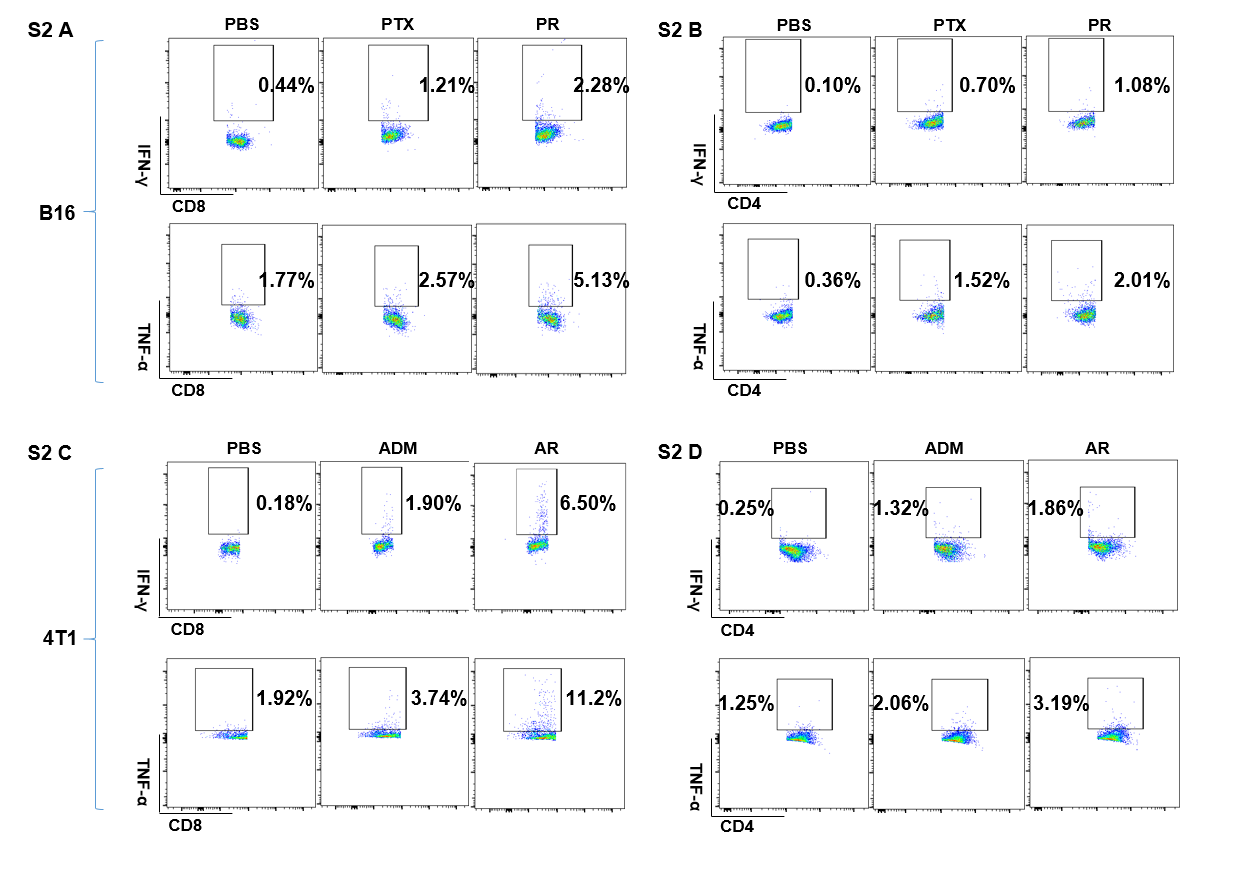


**FIGURE S2 |** Induction of tumor-reactive T cells by local CAER treatment. **(A,B)** Detailed information of flow cytometry for intracellular cytokine (IFN-γ and TNF-α) secretion. Through CD4/CD8 surface and intracellular cytokine (IFN-γ and TNF-α) staining, flow cytometry was performed to analyze the activation of splenic T cells incubated with B16F10 tumor cells in different groups (PBS, PTX, and P+R). **(C,D)** Detailed information of flow cytometry for intracellular cytokine (IFN-γ and TNF-α) secretion. Through CD4/CD8 surface and intracellular cytokine (IFN-γ and TNF-α) staining, flow cytometry was performed to analyze the activation of splenic T cells incubated with 4T1 tumor cells in different groups (PBS, ADM, and A+R).


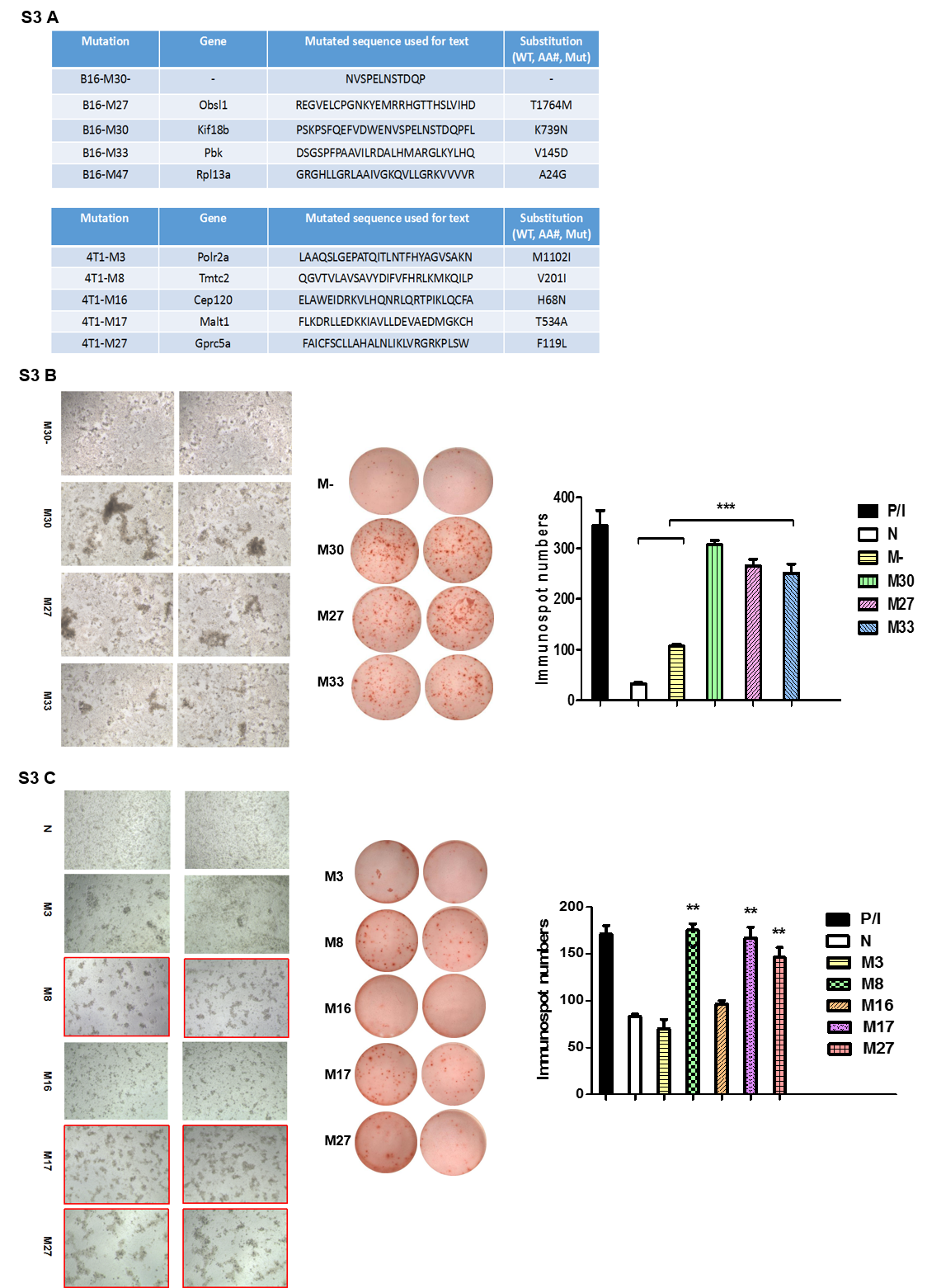

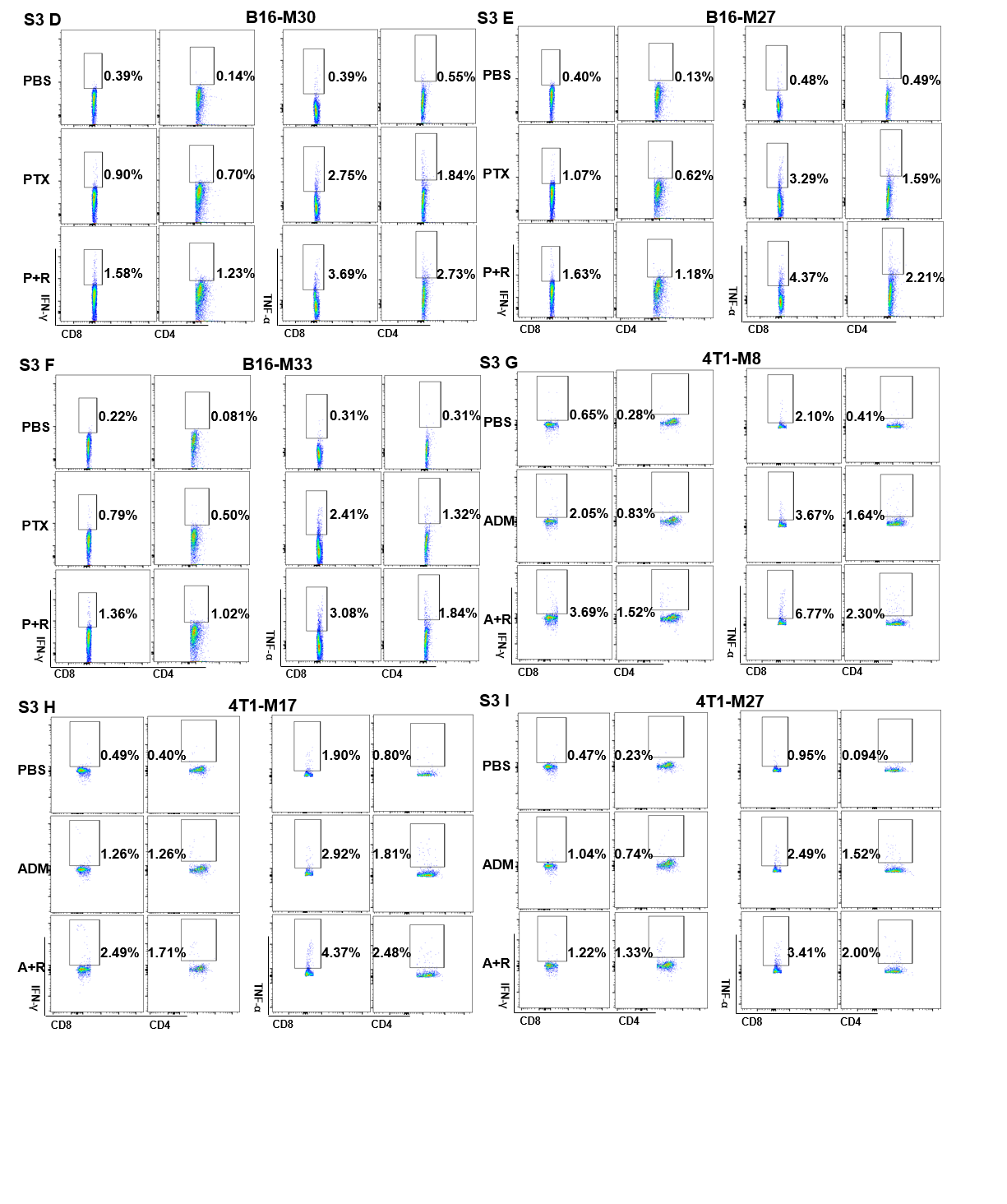


**FIGURE S3 |** Detailed information of B16F10/4T1-associated neoantigen peptides and related immunogenicity testing. **(A)** The concise table showed the detailed synthetic information of B16F10/4T1-associated optimal neoantigen peptides. **(B)** Mature DCs loaded with neoantigen peptides, then incubated with splenocytes from the P+R treatment group of B16F10 tumor bearing C57BL/6 mice. Left panel, the aggregation of mixed lymphocyte reactions (MLR) were observed and imaged under light microscope. Right panel, spontaneous immune responses in splenocytes were tested by ELISpot for recognition of neoantigen peptides or P/I. **(C)** Similarly, spontaneous immune responses in splenocytes from the A+R treatment group of 4T1 tumor bearing BABL/c mice were observed under light microscope and tested by ELISpot for recognition of neoantigen peptides or P/I. **(D–F)** Detailed information of flow cytometry for intracellular cytokine (IFN-γ and TNF-α) secretion in neoantigen specific T cell immune responses of B16F10 model. Through CD4/CD8 surface and intracellular cytokine IFN-γ/TNF-α staining, flow cytometry was performed to analyze the activation of splenic T cells incubated with neoantigen peptides (M30, M27, M33). **(G–I)** Detailed information of flow cytometry for intracellular cytokine (IFN-γ and TNF-α) secretion in neoantigen specific T cell immune responses of 4T1 model. Through CD4/CD8 surface and intracellular cytokine IFN-γ/TNF-α staining, flow cytometry was performed to analyze the activation of splenic T cells incubated with neoantigen peptides (M8, M17, M27). **, P < 0.01; ***, P < 0.001 by Student’s *t*-test.


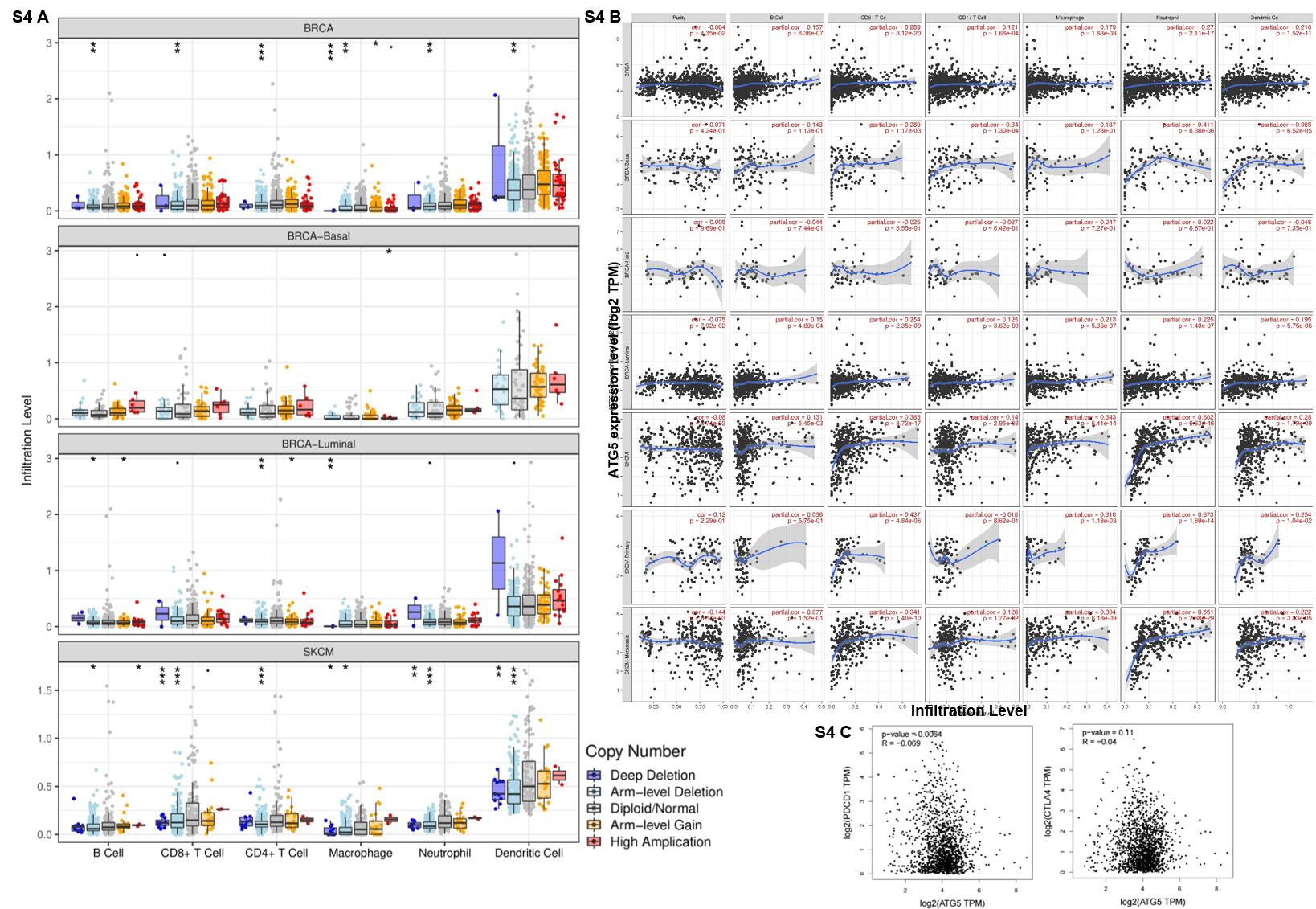


**FIGURE S4 |** The correlations of ATG5 expression with the abundance of six kinds of immune cells in different types of BRCA and SKCM. **(A)** The comparison of immune infiltration levels among different types of BRCA and SKCM tumor tissues with different copy number status of ATG5 gene. **(B)** The correlations of ATG5 expression with the abundance of six kinds of immune cells (CD4^+^ T cells, CD8^+^ T cells, B cells, neutrophils, dendritic cells and macrophages) in different types of BRCA and SKCM tissues. **(C)** The association between ATG5 expression and PD-1 (left panel) and CTLA-4 (right panel) in BRCA and SKCM tumors. *, P < 0.05; **, P < 0.01; ***, P < 0.001.
